# External facilitators and interprofessional facilitation teams: a qualitative study of their roles in supporting practice change

**DOI:** 10.1186/s13012-016-0458-7

**Published:** 2016-07-16

**Authors:** Sylvie Lessard, Céline Bareil, Lyne Lalonde, Fabie Duhamel, Eveline Hudon, Johanne Goudreau, Lise Lévesque

**Affiliations:** 1 HEC Montréal, Montreal, Canada; 2 CETO (Center for Research in Organizational Transformation), Pôle Santé, HEC Montréal, Montreal, Canada; 3 Primary Care Research Team, Centre de santé et de services sociaux de Laval, Laval, Canada; 4 CHUM Research Center (CRCHUM), Université de Montréal, Montreal, Canada; 5 Sanofi Aventis endowment chair in ambulatory pharmaceutical care, Université de Montréal, Montreal, Canada; 6 Faculty of Pharmacy, Université de Montréal, Montreal, Canada; 7 Faculty of Nursing, Université de Montréal, Montreal, Canada; 8 Faculty of Medicine, Université de Montréal, Montreal, Canada

**Keywords:** Facilitation, Facilitation team, Change agent, Roles, Change implementation, Dynamics

## Abstract

**Background:**

Facilitation is a powerful approach to support practice change. The purpose of this study is to better understand the facilitation roles exercised by both external facilitators and interprofessional facilitation teams to foster the implementation of change. Building on Dogherty et al.’s taxonomy of facilitation activities, this study uses an organizational development lens to identify and analyze facilitation roles. It includes a concise definition of what interprofessional facilitation teams actually do, thus expanding our limited knowledge of teams that act as change agents. We also investigate the facilitation dynamics between change actors.

**Methods:**

We carried out a qualitative analysis of a 1-year process of practice change implementation. We studied four family medicine groups, in which we constituted interprofessional facilitation teams. Each team was supported by one external facilitator and included at least one family physician, one case manager nurse, and health professionals located on or off the family medicine group’s site (one pharmacist, plus at least one nutritionist, kinesiologist, or psychologist). We collected our data through focus group interviews with the four teams, individual interviews with the two external facilitators, and case audit documentation. We analyzed both predetermined (as per Dogherty et al., 2012) and emerging facilitation roles, as well as facilitation dynamics.

**Results:**

A non-linear framework of facilitation roles emerged from our data, based on four fields of expertise: change management, project management, meeting management, and group/interpersonal dynamics. We identified 72 facilitation roles, grouped into two categories: “implementation-oriented” and “support-oriented.” Each category was subdivided into themes (*n* = 6; *n* = 5) for clearer understanding (e.g., legitimation of change/project, management of effective meetings). Finally, an examination of facilitation dynamics revealed eight relational ties occurring within and/or between groups of actors.

**Conclusions:**

Facilitation is an approach used by appointed individuals, which teams can also foster, to build capacity and support practice change. Increased understanding of facilitation roles constitutes an asset in training practitioners such as organizational development experts, consultants, facilitators, and facilitation teams. It also helps decision makers become aware of the multiple roles and dynamics involved and the key competencies needed to recruit facilitators and members of interprofessional facilitation teams.

**Electronic supplementary material:**

The online version of this article (doi:10.1186/s13012-016-0458-7) contains supplementary material, which is available to authorized users.

## Background

Facilitation constitutes one of many implementation approaches used to support change within organizations. To help achieve its complex and challenging ongoing transformations, healthcare has shown growing interest in the approach.

Studies, most of which relate to practice facilitation and knowledge translation, reveal various forms of facilitation, with focuses ranging from achieving specific goals (task) to developing processes for better teamwork (holistic) [1]. Over the last few years, healthcare scholars have been increasingly interested in facilitation roles and how they manifest themselves in practice, in order to clarify the concept of facilitation and help practitioners apply it [2–5]. One such study is Dogherty et al.’s [5]. Based on literature and interviews with local and external facilitators following the implementation of evidence-based practice in nursing, they propose a taxonomy that includes 51 facilitation activities divided into 11 groupings, assigned to what the authors call the four stages of facilitation: planning for change, leading and managing change, monitoring progress and ongoing implementation, and evaluating change (see Additional file 1: Facilitation activities performed by appointed facilitators, by Dogherty et al., 2012).

Our study builds on the work of Dogherty et al. to further understand facilitation roles. It was conducted under the umbrella of a larger participatory action research project called Transforming Interprofessional Cardiovascular Prevention in Primary Care (TRANSIT). TRANSIT was implemented in primary care clinics registered as family medicine groups located in greater Laval (Quebec, Canada). Quebec started implementing family medicine groups in 2002 in order to provide citizens with better accessibility to family physicians and improve patient follow-up as well as the quality of primary care services [6, 7]. Each family medicine group is designed to engage physicians in close working collaboration with primary care nurses and other health professionals [7]. The aim of TRANSIT was to improve cardiovascular prevention in primary care patients suffering from multimorbid chronic diseases. It took shape through a three-step process [8]: (1) community-based identification of priorities, informed by the Chronic Care Model [9], (2) design of intervention program and clinical tools [8], and (3) implementation study.

This study focuses on results emerging from the third step, during which two combined facilitation strategies—external facilitation and interprofessional facilitation teams—were tested. Therefore, it not only covers the facilitation roles undertaken by external facilitators (EFs) but also those by interprofessional facilitation teams (IFTs). Every primary care clinic that participated in TRANSIT had to put together an IFT composed of at least one family physician, one case manager nurse, and health professionals located on or off the family medicine group’s site (one pharmacist, plus at least one nutritionist, kinesiologist, or psychologist). IFTs’ main responsibilities were to facilitate implementation and encourage each discipline to take ownership of change, while the EFs’ were to facilitate team meetings and provide support to IFT members.

Our work differs from Dogherty et al.’s research in important ways. It builds general empirical knowledge about facilitation roles by (1) using an organizational development lens to identify and analyze the specific facilitation roles undertaken by EFs and IFTs, (2) targeting IFTs in its study objectives, (3) utilizing data that emerged not only from facilitators but also from facilitated group members, in order to document the facilitation roles practiced by both types of change agents, and (4) testing the application of Dogherty et al.’s taxonomy in another setting.

Furthermore, although studies abound on implementation efforts that involve teams to facilitate change throughout organizations, we have found (as have others) that “there has been no empirical organizational behavior research on these kinds of … teams” ([10]:369). Thus, there is a need to examine what facilitation teams actually do in practice to support change.

Finally, authors have recently acknowledged that more research is needed to refine current approaches [11], to examine how quality improvement interventions work [12] and to further develop our understanding of the facilitation approach [13]. Therefore, this study focuses not only on the identification of facilitation roles but also on the dynamics of facilitation.

### The concept of facilitation and “roles”

Definitions of facilitation emphasize different characteristics of the concept. For example, we have facilitation as the role of a single individual [14–16], facilitation as a process [3, 16, 17], and facilitation aiming at helping a group of people [16, 18]. Facilitation is also related to the concept of change agent, defined as “an internal or external individual or team responsible for initiating, sponsoring, directing, managing or implementing a specific change initiative, project or complete change programme” ([19]:139). In fact, there are multiple examples showing how different categories of change agents (such as leaders, managers, management consultants, and teams [19]) have been associated with facilitation [5, 15, 20–22]. This study focuses on the facilitation roles performed by two types of change agents: consultants or EFs, and teams.

But what does the word “role” actually mean? Authors’ views vary: it can refer to characteristic behaviors, to social parts to be played, or to scripts or expectations for social conduct [23]. In the facilitation literature, terms such as competences, dimensions, functions, and tasks [24–35] are used to describe expectations or what a facilitator should be doing. Role behaviors are described by using terms such as activities, actions, behaviors, interventions, or impact codes [3, 5, 25, 29, 30, 36–40]. For this study, the term “role” is the most appropriate, as it encompasses observed behaviors as well as expectations related to EFs and IFTs.

### Study purpose and objectives

The overall purpose of this study is to enhance our understanding of the roles exercised by EFs and IFTs to support practice change implementation in organizational contexts. More specifically, this qualitative research is guided by the following objectives:

1. identifying and analyzing the facilitation roles undertaken by EFs and IFTs during the implementation of TRANSIT
2. examining the dynamics of facilitation between EFs, IFTs, family medicine groups, and other change actors

## Methods

### Participants and sites

Our study involved four family medicine groups, each represented by an IFT. The IFT concept was created as a temporary structure by the research team, for the duration of the implementation phase (12 months). In view of the number of clinicians in family medicine groups who had to be reached (between 10 and 46), each IFT was expected to fulfill four key responsibilities: (1) to act as a liaison to encourage each discipline to take ownership of change, (2) to select at least one of six TRANSIT interventions to be implemented in the family medicine group [8] (i.e., coordination of interprofessional follow-up by primary care nurse-case manager; case manager referrals to public group classes or private health professionals; clinicians’ training and usage of motivational interviewing; utilization of patient-health booklet; application of collective prescriptions; utilization of internet-based directory of community and health resources), (3) to develop action plans accordingly, and (4) to translate knowledge and disseminate change across the family medicine group and other external health specialists. Each IFT was constituted of at least one family physician, one case manager nurse, and health professionals located on or off the family medicine group’s site (one pharmacist, plus at least one nutritionist, kinesiologist, or psychologist). Some included administrative assistants.

Two EFs were hired by the research team. Each worked part-time, on a 2.5 days per week basis. One was a nurse with a master’s degree in health administration; the other one was an experienced pharmacist with background and knowledge in academic detailing and project management. Each EF was randomly assigned to two IFTs. During the 8 months preceding TRANSIT’s implementation, researchers (CB and JG) provided EFs with training on facilitation, change management, project management, PDSA methodology, interprofessional collaboration, primary care services in clinics, Chronic Care Model, and the TRANSIT program.

### Data collection

This study is based on multiple sources of data, which adds strength to the evidence it provides [41]. Empirical data was obtained through interviews with EFs (*n* = 4 times 2 EFs) and IFT members (*n* = 2 times 4 IFTs), as well as in-depth analysis of case audit documentation. Table 1 shows the numbers of data collection participants, interviews, and documents, as well as periods of data collection.

**Table 1:** Data sources

Data collection method	Participants	Total interviews	Total reviewed documents	t3	t6	t9	t12
Individual interviews with EFs	2 EFs	8	–	√	√	√	√
Interviews with IFTs	4 IFTs	8	–		√		√
Meeting minutes from IFT meetings	32 IFT members	–	37	---- Ongoing ----			
Field notes/reports from EFs	2 EFs	–	55 + 37	---- Ongoing ----			

The research team developed semi-structured interview guides. For quarterly individual interviews with EFs, the guides included open-ended questions about their experience, what went well, and what was more difficult in their facilitator role; their concerns as an EF; and their experiences and perceptions of the IFTs and their roles. These interviews lasted 1 h each. Interview guides for semestrial focus group interviews with each IFT included open-ended questions about their experience of facilitation, their facilitation team, their EF, as well as their experience of the TRANSIT program, what was going well, and what was more challenging. For the final group interviews, a question about the sustainability of TRANSIT was added. Interviews with IFTs lasted 2 h each. All EFs and IFT interviews were digitally recorded, with permission, and transcribed externally.

Case audit documentation comprised three kinds of documents: minutes from all formal IFT meetings (*n* = 37), logs from EFs about each IFT meeting (*n* = 37), and EFs’ field notes (*n* = 55). Written logs created by EFs after each formal IFT meeting included data related to their perceptions and observations about the meeting, challenges encountered and solutions, facilitative aspects, facilitation tools and strategies used, concerns brought up by IFT members, role-sharing among the team, interpersonal relationships, meeting evaluation, and lessons learned. EFs wrote digital field notes to record events or activities in which they participated outside formal meetings, indicating date, family medicine group identification, event/activity description, and comments/perceptions/results.

Using only primary data (i.e., data from interviews) would have limited our evidence to participants’ own perceptions and comments. By bringing in additional secondary data included in case audit documentation, we could complement and illuminate the information obtained through primary data, as documents were written sooner after events or meetings. Minutes also informed us about facts, role assignments, and decisions made by IFTs during the implementation—details that might not have been captured during interviews.

### Data analysis

As described in the following paragraphs, our analysis followed Miles et al.’s qualitative analysis framework, which is composed of three concurrent activity flows: data condensation, data display, and drawing and verifying conclusions [42].

In order to condense the data and identify facilitation roles undertaken by EFs and IFTs, we applied a process-coding method, which involves using “gerunds (“-ing” words) … to connote observable and conceptual action in the data” ([42]:75). Therefore, all codes used to identify facilitation roles were formulated starting with a gerund (e.g., encouraging, evaluating, assisting). We provisionally listed 51 codes, based on Dogherty et al.’s taxonomy of facilitation activities [5]. Attribute codes were created to identify the speaker, the data source, who performed each role (EF or IFT), and the family medicine group involved.

To increase the reliability and clarity of code definitions, two researchers (SL, LLe) separately coded 6-month interview transcripts with qualitative analysis software (*QDA-Miner* 4.1.3). In the beginning, but also during the coding, they discussed with another researcher (CB) and agreed the meaning of codes deduced from Dogherty et al.’s model (in which the descriptions of facilitation activities were not always clear), as well as emergent codes. Multiple process codes per datum were permitted, since a particular datum might relate to more than one role. The EF facilitation role was defined as an activity or action that at least one EF had undertaken during the 1-year implementation period of TRANSIT, while the IFT facilitation role was defined as an activity or action that at least one member of the IFT (other than the EF) had put forward in order to facilitate the implementation of the project or change.

We amended certain role descriptions used in Dogherty et al.’s taxonomy of facilitation activities, for two reasons. First, we wanted to make role descriptions more generalizable, so they would not only apply to a nursing or research context. Accordingly, we modified role descriptions that referred specifically to practice facilitation, knowledge utilization, research, or healthcare (e.g., “highlighting a need for practice change” became “highlighting a need for change”). Second, some of Dogherty et al.’s activities needed clarification because they caused confusion during coding. For instance, we split their activity called “performing/assisting with evaluation” into two different roles, one aimed at evaluating implementation aspects, the other aimed at evaluating group meetings.

Case audit documentation and later interview transcripts were coded by the main author, based on the same codebook. Codes that subsequently emerged were discussed and agreed upon with at least one other researcher.

Coded data was displayed in an *Excel* matrix for review and sub-coding, thus attaining greater precision in terms of role meaning. To draw and validate conclusions, we identified patterns and referred to facilitation-related literature, which helped us categorize facilitation roles and develop an emergent conceptual framework. By reviewing sub-codes, we could identify the actors at whom each role was aimed and determine the relationships between them when facilitation roles were exerted, thus characterizing the dynamics involved during the facilitation process.

## Results

From February 2012 to February 2013, each IFT held between eight and ten 2-h meetings, supported by one EF for the implementation of TRANSIT. Ongoing coaching was provided to EFs by researchers and occurred between EFs throughout implementation. The following presents the results of our study, guided by the aforementioned study objectives.

### A framework of facilitation roles by fields of expertise: objective #1

Although IFTs saw facilitation as being mainly related to meeting management, guidance towards objectives, and support, facilitation roles encompassed a broad spectrum of other activities. We identified a total of 72 facilitation roles undertaken by EFs and/or IFTs during the implementation of TRANSIT, which we have organized into a new framework of facilitation roles. Our rationale for this new framework is discussed later.

**Fig. 1:**
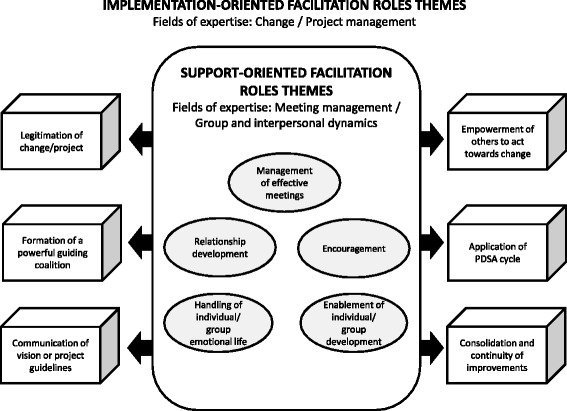
Categories and themes of facilitation roles. This conceptual framework of facilitation roles offers an overview of facilitation categories and themes as well as the relationship between implementation-oriented facilitation roles and support-oriented facilitation roles

Our framework divides facilitation roles into two main focus-oriented categories: (a) implementation-oriented facilitation roles and (b) support-oriented facilitation roles. This categorization accords with the mindset adopted by other scholars, who have found facilitation activities that were oriented towards “meta-support for implementation,” “support to individual practices,” and “group support to individual or/and practices” [39]. Our two categories complement each other in order to help organizations reach change/project implementation goals. Figure 1 provides an overview of facilitation categories and themes, as well as the relationship between implementation- and support-oriented facilitation roles, assuming that support roles serve as a basis to sustain implementation roles.

Facilitation involves roles from four fields of expertise. Implementation-oriented facilitation roles relate to roles in change management (i.e., models such as Kotter’s eight-step process for leading change [43] and Young’s meta model of change [44]) and roles in project management (i.e., models such as the PMBOK guide [45]). Since the fields of change management and project management both refer, one way or another, to the importance of meeting management and taking care of group process through change/project implementation, we believe that support-oriented facilitation roles are somewhat embedded within implementation roles. Nevertheless, we present them separately to enable a better understanding of the facilitating agents’ focus and roles. Support-oriented facilitation roles therefore relate to roles in the fields of meeting management and group and interpersonal dynamics, which aim at supporting group effectiveness, as well as supporting individuals or teams during the implementation of a change/project.

Models from these fields of expertise have helped us interpret the data and identify subcategories or themes emerging from our examination of patterns during data analysis. Six different themes emerged in the “implementation-oriented facilitation roles” category and 5 in the “support-oriented facilitation roles” category, for a total of 11 themes. Most subcategories include facilitation roles that combine knowledge related to two fields of expertise (change and project management or meeting management and group/interpersonal dynamics), but some roles are more specifically related to one field or another. Additional file 2 shows all 72 facilitation roles by category and theme and how they relate to fields of expertise. Also, theme definitions and some evidence (verbatim or text from documentation) from the TRANSIT study are available (see Additional file 3: Definitions and evidence related to facilitation roles themes).

Of the 72 facilitation roles identified through our empirical data analysis, a few were performed only by EFs, others only by IFTs (see Table 2: Facilitation roles undertaken exclusively by EFs and IFTs). The rest were performed by both.

**Table 2:** Facilitation roles undertaken exclusively by EFs and IFTs

Nine roles exclusive to EFs	Three roles exclusive to IFTs
• Providing skills training	• Using storytelling
• Stimulating critical inquiry and assisting groups to develop/refine specific project-related questions	• Discussing specific cases/experiences within the scope of the project
• Thinking ahead in the process	• Linking implementation actions to outcomes
• Performing meeting evaluations	
• Tailoring/adapting facilitation services to the local setting	
• General administrative planning	
• Listening actively, clarifying and summarizing the information	
• Observing group members’ behaviors	
• Sharing benchmarking results from multiple sites to encourage team	

Finally, even though a certain sequence in the themes of implementation-oriented facilitation roles may seem logical, the roles within these themes did not always follow a linear process. For instance, EFs or IFTs had to take on roles related to the legitimization of change/project at different times during implementation. As for the themes of support-oriented facilitation roles, they include roles that were simply undertaken as and when they were needed.

### Facilitation dynamics: objective #2

Another of our findings relates to the dynamics of facilitation—in other words, the social interactions between the actors involved in the facilitation process. All along the preparation and implementation of TRANSIT, facilitation was presented to the participants as a one-way process that flowed from the EF towards each IFT and then on to their family medicine group and related external clinicians. However, our data analysis revealed another scenario (see Additional file 4: Facilitation roles by change agent and relationships involved in facilitation).

We found that five actors or groups of actors were involved during TRANSIT’s facilitation process: (1) the research group, composed of researchers and administrative staff who issued the research protocol and managed clinical and research administration guidelines (e.g., indicators, compensation policies, and budget allocations); (2) the two EFs, assigned and trained by the research team; (3) the IFTs, a multidisciplinary team of healthcare professionals; (4) the organization, comprising all physicians, clinicians, and administrative staff working on site at the family medicine group’s location, affected by changes related to TRANSIT but not participating in the IFT; and (5) other external change agents, including actors not based at the family medicine group’s location (i.e., clinicians to whom patients were referred to, community pharmacists, representatives of health agencies, governmental entities, and patient education groups such as diabetes training groups).

**Fig. 2:**
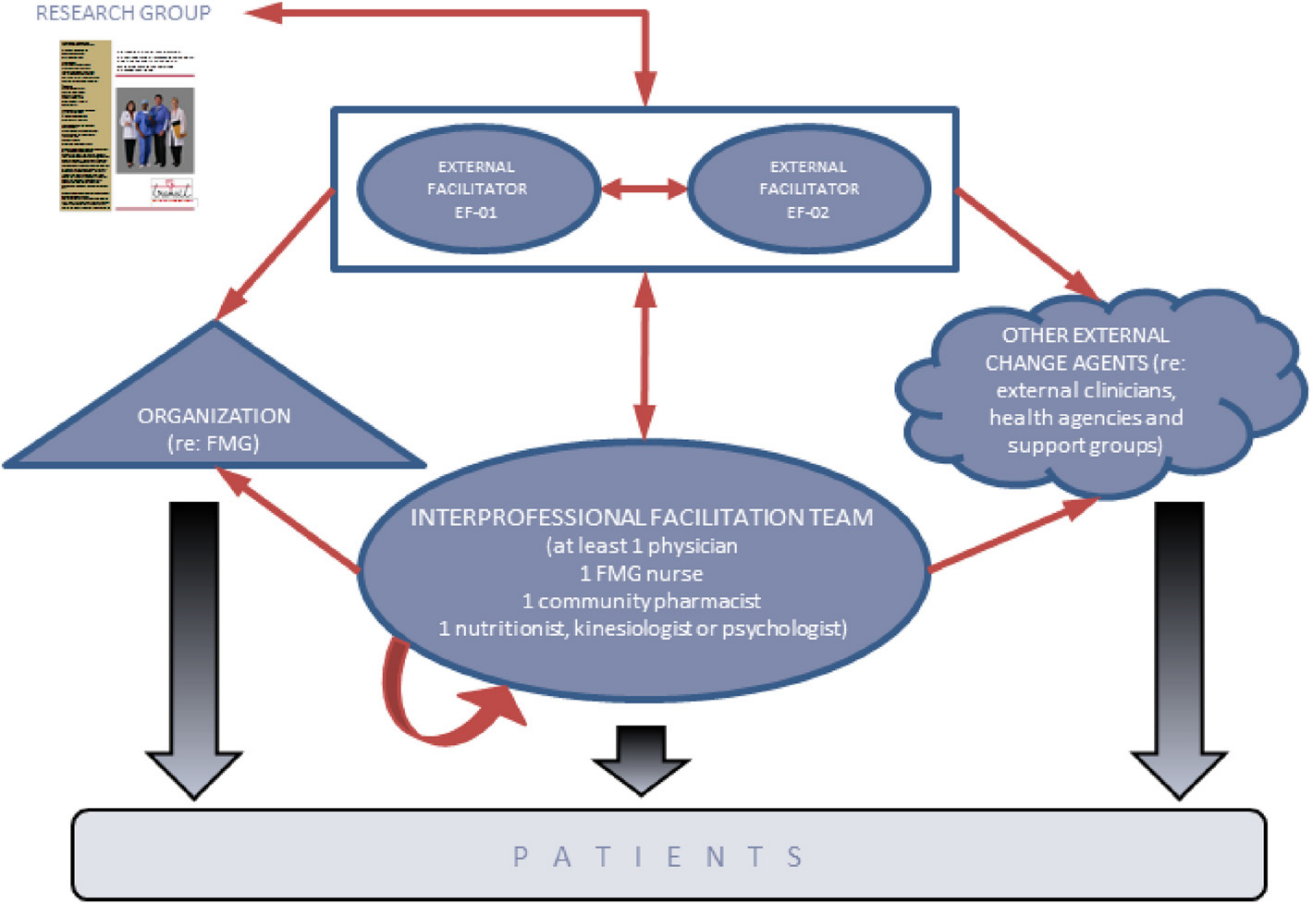
Facilitation dynamics in TRANSIT study. Facilitation involves dynamic interrelations between groups of actors and/or between actors from a same group, as shown by the red arrows (*n* = 8) in this figure

We identified eight relational ties or dynamic relations, supported by behavioral interactions [46] depicting facilitation roles between these groups of actors or between actors from a same group (represented by the red arrows in Fig. 2).

Our data analysis showed that facilitation roles followed bidirectional (reciprocal) routes between actors involved in project/change management (research group, EFs, IFTs) and unidirectional routes towards the organization and other external change agents. Facilitation roles were also undertaken by IFT members in order to help their own team. Table 3 shows examples of facilitation roles with related evidence for each type of social interaction, as well as the actor towards whom facilitation was oriented.

**Table 3:** Examples of social interactions during TRANSIT's facilitation process

Social interaction	Examples of facilitation roles	Related datum
Research group facilitating towards EF	Providing ongoing support/reassurance and constructive feedback	“… we have contacts in the team, which helps. … [research agent 1] helps me a lot. [Research agent 2] does too, with her advice on administration aspects.” (Interview T3-EF2)
EF facilitating towards Research group	Acting as a liaison	“… she [external facilitator] really served as a transmission belt with the research team. … More concretely, … [finding out about] budget utilization, rules, how to use them, how to bill. Questions that we had about the clientele, if we had the right to know some information … about our patients” (Interview T12-Physician-IFT1)
EF facilitating towards another EF	Providing ongoing support/reassurance and constructive feedback	“… With [external facilitator 1], we talk to each other almost every day. We encourage each other. … After our meetings we always call each other to discuss what happened. [External facilitator 1] calls sometimes, also, to say: it didn’t go too well in this meeting, what do you think?… And it’s the same thing with me.” (Interview T3-EF2)
EF facilitating towards IFT	Helping to build in the structures/processes to support staff and help them overcome obstacles	“… there are three of them, they send each other a lot of documents… because I saw it over the long term, of course: there are documents where they can enter data, but other people will enter other data, so there is a risk of errors, etc., so I proposed a way of working together…” (Interview T9-EF2)
EF facilitating towards organization (family medicine group)	Increasing awareness of and helping overcome resistance to change	“I explained that it’s normal if they don’t feel they’re on the same level as [the nurse on the IFT]. I highlighted the importance of the case management nurse. [The IFT nurse] had suggested that I discuss the following items with them: talk about the indicators… that most indicators are covered if they use the tools provided by TRANSIT… (EF Field notes T9-EF1-on FMG4)
EF facilitating towards other external change agents	Meeting actors of change outside regular meetings	“…we also had a meeting with the nutritionists, which we organized in June… Also on that day, we had the pharmacists, the family medicine group pharmacists …” (Interview T6-EF1)
IFT facilitating towards EF	Providing feedback about implementation	“… it’s the nutritionist who keeps me informed mostly, who calls me and says: I spoke with [the kinesiologist], I spoke with [the nurse]… these are the problems…” (Interview T9-EF2)
IFT facilitating towards IFT	Discussing specific cases/experiences within the scope of the project	“What is interesting and new at this fourth meeting, it’s that there are case discussions quickly brought up and presented by [the nurse] informally—i.e. it is integrated within discussions planned in the agenda—others propose ideas – solutions.” (EF Field notes T6-EF2 on IFT3)
IFT facilitating towards organization (family medicine group)	Creating an open, supportive, and trusting environment conducive to change	[About receptionists/administrative assistants giving access to patient files:] “Int: So there is no resistance in the clinic at this level, like people saying, ‘Well, who are you to take my file?’ Kinesiologist: We introduced ourselves to the receptionists.” (Interview T6-Kinesiologist-IFT3)
IFT facilitating towards other external change agents	Advocating for resources and change	“About external community pharmacists… those under my commercial banner, many have approached me… to get details, but still, it’s all about communication…” (Interview T6-Pharmacist-IFT4)

## Discussion

Our findings allow us to attest that facilitation is a dynamic strategy used to support change implementation. In this context, it involves the use of various facilitation roles related to four fields of expertise (project management, change management, meeting management, and group and interpersonal dynamics) that are oriented towards project/change implementation as well as support. Individual and group change agents can undertake facilitation roles, although certain roles are more likely to be undertaken by an EF or an IFT. Also, the dynamics of facilitation reveal reciprocal interactions between change actors. Therefore, individual and team change agents who undertake facilitation roles may be influenced by facilitation roles that other change actors may be undertaking.

In the following sections, we present the four key learnings gained from our new understanding of facilitation roles: (a) the importance of a clear understanding of the various categories of facilitation roles, (b) the non-linearity of facilitation role subcategories, (c) the expansion of knowledge on facilitation teams, and (d) the recognition of stakeholders as actors of the facilitation process.

### Understanding the various categories of roles involved in facilitation

According to our findings, facilitation relates to four fields of expertise. First, many authors refer to facilitation as a change agent strategy [1, 3, 5, 19, 47, 48], thus connecting facilitation with the field of change management. Second, the link between project management and facilitation has also been established [3, 5, 38, 49]; one EF in our study even observed that “the two go hand in hand.” Third, studies on the facilitation of computer-supported meetings [17, 25, 50–53] as well as numerous guidelines or articles on facilitation [30, 33, 54] have demonstrated the relationship between facilitation and meeting management. Finally, the fact that certain facilitation experts [15, 28] have based their facilitation frameworks on group efficiency models supports the link between facilitation and the field of group and interpersonal dynamics.

Increasing the body of knowledge on facilitation, several facilitation roles emerged from our study, in contrast with Dogherty et al.’s taxonomy [5]. There could be various reasons for this. First, we found several roles (e.g., meeting actors of change outside regular meetings, establishing political links with/influencing stakeholders, reporting/managing conflict, establishing ground rules) that complemented or clarified Dogherty et al.’s facilitation activities. Second, since TRANSIT was a multisite project in which each EF was assigned to more than one site, the project design itself may have led to the emergence of facilitation roles (e.g., sharing ideas across project sites). Third, coaching provided by the research team to EFs could have led them to undertake certain facilitation roles (e.g., reflecting/planning on the “after-project” to consolidate improvements and institutionalize changes). Finally, the fact that IFTs included primary care professionals who were also involved in dealing directly with TRANSIT patients may have triggered the identification of “discussing specific cases/experiences within the scope of the project” as a facilitation role—one that is particularly relevant for implementing change within primary care practices, but still applicable to other contexts.

Although most of our emergent roles could be found in literature, sometimes with different wording, three had not been mentioned previously as facilitation roles: discussing specific cases/experiences within the scope of the project, providing feedback about implementation, and reflecting/planning on the “after-project” to consolidate improvements and institutionalize changes.

In practice, it can be difficult to know where one’s facilitation role ends. Our framework can be used as a reference to set the limits of the facilitation roles. For instance, although it has been recognized that the facilitator role can range between “doing for others” and “enabling others” [1], it is easy to cross the line into doing too much, and an EF may be asked to directly intervene or communicate with patients (as in fact occurred during TRANSIT). The wise EF preserves some distance from day-to-day tasks, since being too hands-on would not be helpful in the long term. According to our framework, the facilitation role described as “taking on specific tasks” should be confined to bounded tasks related to implementing change. However, good judgment and coaching would also help the facilitator in need of guidance.

Our framework, composed of fields of expertise, main categories, themes, and roles of facilitation, brings a clearer understanding of what facilitation encompasses. It can be used to clarify expectations towards a facilitating agent (e.g., roles expected of EFs and IFTs) or serve as a training tool for EFs and IFTs. Because groups often do not know what to expect from an EF (as reported within our study), the EF may gain credibility by explaining to a group that facilitation includes roles oriented towards the implementation of the project or change, as well as roles oriented towards support, giving examples for each category. It would also be relevant, at the start of a change project, to present the IFT with the different roles that team members might be expected to undertake as facilitators throughout project/change implementation.

More research is needed to test our framework, especially with respect to facilitation teams, to help define the contextual aspects that support certain facilitation roles more than others, to understand how leadership is shared during the facilitation process, and to evaluate how the composition of facilitation teams may influence the undertaking of facilitation roles (e.g., including patients as members of facilitation teams). Researchers should be aware that such studies can be very complex, expensive (especially if participants are compensated), and require the abilities, resources, and time needed to reach interrater agreement and thoroughly analyze large amounts of qualitative data.

### Non-linear subcategories of facilitation roles

Certain facilitation roles might be expected to take place at the start or at the end of a meeting or change project. However, our findings indicate that they do not follow a linear process during implementation and that facilitation involves making decisions in the moment [40]. Therefore, we do not see any particular order in the subcategories or themes of facilitation roles, in contrast to Dogherty’s et al. facilitation steps (planning for change, leading and managing change, monitoring process and ongoing implementation, and evaluating change) [5]. Many scholars have criticized linear change models such as Lewin’s three-step model (unfreezing, changing, refreezing), on which facilitation models such as Dogherty et al.’s seem to be built. As one of them, Pettigrew has maintained that “change is not a continuous incremental process” ([55]:1307) and that political and cultural factors affect change outcomes. Complexity theories have fueled the debate, considering organizations as dynamic non-linear systems [56]. Practitioners have also recognized the important overlap and interpenetration between change stages [57]. Our observations lead us to agree with these perspectives.

As the TRANSIT study was a dynamic program, it may have allowed IFTs to select the interventions they wanted to implement at each site, without following any preplanned process. Also, as primary care constitutes a very complex setting that is undergoing multifarious and continuous change, flexibility and adaptability are required all along from individuals and teams performing facilitation. More research is needed to define the contextual aspects related to facilitation roles and why they are undertaken at certain moments.

### New knowledge on facilitation teams

Empirical knowledge about teams acting as change agents is limited [10, 19, 58] and even less is known about what facilitation teams (sometimes called change teams or implementation teams) actually do. Dogherty et al. have pointed out that facilitation is not only just an appointed role but also a process that can be shared between team members and an appointed facilitator [5]. They note that the following activities were performed by team members throughout their study: identifying a leader, highlighting a need for practice change, selecting an area relevant to staff/recognized as a priority, performing a practice audit, and helping to interpret the research and applying it in practice. In comparison, we found 63 facilitation roles that were performed by IFTs. Identifying and categorizing these numerous and varied roles helps to expand our concrete knowledge of what facilitation teams do in practice.

In examining our results, we found nine facilitation roles that were not undertaken by IFTs, only by EFs. They were mostly concerned with consulting and monitoring group process, which speaks to the advantages of using an external resource to help an IFT accomplish its goals (e.g., expertise and knowledge, external perspective to the group, able to focus on group processes [35, 59, 60]).

Furthermore, our study elucidates the interactions involved when an IFT or a team member engages in facilitation. IFT roles require interacting within the team’s own organization (family medicine group), with external change actors (external clinicians, health agencies or governmental representatives, support groups), and with the EF. Some facilitation roles may even be directed from a team member towards their fellow team members or to the team as a whole. These facilitation dynamics therefore extend the responsibility for facilitation efforts beyond the facilitating change agents themselves. Individuals who join as IFT members, as well as immediate supervisors and decision makers, should be aware that facilitation involves such interactions, which may require certain abilities, credibility, and dedicated time and efforts.

More research is needed to examine the facilitation roles undertaken by IFTs under different contexts and to identify the relative impact of these roles.

### Recognizing stakeholders as actors of the facilitation process

Stakeholders are the end users of the knowledge being implemented in practice development programs such as TRANSIT [61]. As such, they should be considered as important actors of the facilitation process, even more so since their positive perception of the change/project has already been recognized as one of the key factors for successful projects/changes [62–64].

McCormack and Garbett have briefly presented some of the interactions involving stakeholders in practice development activities, which included relationships with individuals, teams, or larger groupings within or outside the organization (practitioners, middle and senior level managers, representatives from other healthcare occupations or user groups) [29]. Our results also demonstrate that certain facilitation roles are aimed at influencing the perceptions of stakeholders such as the IFT, of people working for the organization where the change/project is taking place (e.g., family medicine group), and of other external stakeholders (e.g., external clinicians, health agencies or governmental representatives, support groups).

Change agents who perform facilitation would benefit from understanding and managing with the various types of stakeholders, especially when politics and complexity loom as large as they do in healthcare. Although introducing evidence-based knowledge to improve the delivery of preventive health could be seen as a purely technical change, it also encompasses strategic, cultural, and structural dimensions [65]. The complexity of the primary care team, regarded as a complex adaptive system [65, 66], should also be considered. Certain facilitation roles aimed towards change actors within the organization, or other external stakeholders, might be more appropriate depending on the type of stakeholder they are aimed at. Stakeholder theory could be used to help identify the types of stakeholders involved (e.g., stakeholders can be categorized based on their power, legitimacy, and/or urgency [64, 67]). Further studies on how facilitation roles can influence certain types of stakeholders would enlighten facilitation practitioners on the roles they should favor depending on the situation.

### Study limitations

This study was based on a 3-year research project aimed at implementing evidence-based practices in primary care over a 1-year period. It relies on the experience of two EFs and four IFTs, in four family medicine groups located in Quebec, Canada.

The fact that TRANSIT used only two EFs may be seen as a limitation to accessible data. However, the data collected through interviews and case audit documentation over the course of 1 year was very rich. It actually allowed us to find all and more than Dogherty et al.’s 51 facilitation activities, which reinforces the validity of our results.

The particular context in which our study was conducted—research program, quality improvement, participatory, project implementation, and knowledge translation—may have led us to identify certain facilitation roles that may not be present in other organizational contexts where facilitation is used (e.g., conflict resolution, team-building, training). The duration of our project may also have hindered our results, as many IFTs did not have enough time to fully implement TRANSIT within a year, which meant that some facilitation roles identified in the literature were not undertaken. Nonetheless, we believe that even though some of the specific roles we identified may only appear under certain conditions (e.g., reporting/managing conflict, storytelling), the themes that were determined in our framework may apply to other change/quality improvement implementation projects that use facilitation as a strategy.

We did not assess the relative importance of each role in terms of implementation (e.g., some roles may have been more predominant than others), nor the relative importance of the roles undertaken by EFs in comparison with those undertaken by IFTs. The challenges encountered by EFs and IFTs in performing these roles have not been addressed either. These elements could be taken into account in future research.

Finally, our search for facilitation roles was primarily based on Dogherty et al.’s framework, since it appeared to be the most complete and appropriate tool for analyzing the data we collected during TRANSIT, an evidence-based program related to practice development and nursing. Our initial codebook of facilitation roles might have been more complete if we had based it on a more extensive and systematic literature review on facilitation within organizational contexts. Nevertheless, the fact that we validated our findings based on the literature on facilitation roles in healthcare, as well as in other organizational contexts, has helped bring more clarity to our proposed framework.

## Conclusions

Facilitation is an approach that can be used by individual and team change agents to help build capacity and support practice change within organizational contexts. It encompasses a broad array of roles, oriented towards both implementing change and supporting individuals or groups.

We present a framework of facilitation roles based on fields of expertise that expands the concept of facilitation beyond practice facilitators to a larger group of users. Because of its format, and the way in which it simplifies the different aspects of facilitation, our framework could be used to train and support the work of organizational development experts, consultants, appointed facilitators, and facilitation teams involved with the implementation of organizational change. It could also be used to increase decision makers’ awareness of the diversity of facilitation roles that change agents may be undertaking in a project or change program, as well as the skills required for recruiting.

## Abbreviations

EF, external facilitator; IFT, interprofessional facilitation team; TRANSIT, Transforming Interprofessional Cardiovascular Prevention in Primary Care

## Additional files

Additional file 1: Facilitation activities performed by appointed facilitators, by Dogherty et al., 2012. PDF file displaying Dogherty et al.’s taxonomy of facilitation activities, which served as a basis for analysis. (PDF 18 kb)

Additional file 2: Facilitation role categories and roles, by fields of expertise. PDF file displaying all 72 facilitation roles identified in this study, by category and theme, and how they relate to four fields of expertise (change management, project management, meeting management, and group/interpersonal dynamics). (PDF 361 kb)

Additional file 3: Definitions and evidence related to facilitation roles themes. PDF file displaying descriptions and definitions of facilitation themes, as well as illustrating excerpts from TRANSIT study. (PDF 249 kb)

Additional file 4: Facilitation roles by change agent and relationships involved in facilitation. PDF file displaying facilitation roles undertaken by EFs and by IFTs, as well as change actors involved for each facilitation role. (PDF 181 kb)
