# Supplementary material for: External facilitators and interprofessional facilitation teams: a qualitative study of their roles in supporting practice change
Source: Implement Sci. 2016 Jul 16;11:97. doi: 10.1186/s13012-016-0458-7 (PMC4947272; doi:10.1186/s13012-016-0458-7)
Supplement: Supplementary file 1 — Facilitation activities performed by appointed facilitators, by Dogherty et al., 2012. PDF file displaying Dogherty et al.’s taxonomy of facilitation activities, which served as a basis for analysis. (PDF 18 kb) [file 13012_2016_458_MOESM1_ESM.pdf]

ADDITIONAL FILE 1:

Facilitation activities performed by appointed facilitators, by Dogherty *et al*., 2012

Lessard S, Bareil C, Lalonde L, Duhamel F, Hudon E, Goudreau J, Lévesque L: **External facilitators and interprofessional facilitation teams: a qualitative study of their roles in supporting practice change**

**Planning for change**	
Increasing awareness<br>1) Highlighting a need for practice change<br>2) Selecting an area for change relevant to staff/recognized as a priority<br>3) Stimulating critical inquiry and assisting groups to develop/refine specific clinical practice questions<br>4) Assisting with/performing a formal/informal practice audit<br>5) Interpreting baseline data and providing feedback/insight into performance gaps<br>6) Emphasizing enhanced patient outcomes as opposed to poor practice as reason for change	*Developing a plan*<br>7) Goal-setting and assisting with development of an action plan<br>8) Helping identify and determine solutions to address potential barriers to EBP<br>9) Displaying and generating enthusiasm at the start of the project<br>10) Thinking ahead in the process
**Leading and managing change**	
Knowledge and data management<br>11) Knowledge translation/dissemination (assisting with conducting literature searches, obtaining articles, appraising and summarizing the evidence)<br>12) Helping to interpret the research and apply it in practice<br>13) Providing resources/tools for change	*Project management*<br>14) Identifying a leader<br>15) Establishing and allocating roles/delegating responsibilities<br>16) Advocating for resources and change
Recognizing the importance of context<br>17) Creating an open, supportive, and trusting environment conducive to change<br>18) Helping to build in the structures/processes to support staff and help them overcome obstacles<br>19) Creating local ownership of change<br>20) Assisting with adapting evidence to the local context<br>21) Boundary-spanning (addressing organizational systems/culture), managing the different requirements of each discipline/role<br>22) Tailoring/adapting facilitation services to the local setting	*Fostering team-building/group dynamics*<br>23) Relationship-building<br>24) Encouraging effective teamwork<br>25) Enabling individual and group development<br>26) Encouraging/ensuring adequate participation<br>27) Increasing awareness of and helping overcome resistance to change<br>28) Consensus-building (shared decision-making)
Administrative and project-specific support<br>29) Organizing/scheduling meetings<br>30) Leading/participating in meetings<br>31) Gathering information and assembling/distributing reports and materials	32) General planning<br>33) Providing skills training<br>34) Taking on specific tasks
**Monitoring progress and ongoing implementation**	
Problem-solving<br>35) Problem-solving and addressing specific issues<br>36) Making changes to the developed plan as necessary<br>37) Networking	*Providing support*<br>38) Mentoring and role-modelling EBP<br>39) Maintaining momentum and enthusiasm<br>40) Acknowledging ideas and efforts<br>41) Providing ongoing support/reassurance and constructive feedback<br>42) Empowering group members<br>43) Providing advice/guidance/assistance<br>44) Being available as needed<br>45) Ensuring group remains on task and things are not missed (process/methodology is followed)
Effective communication<br>46) Providing regular communication (emails, phone calls)<br>47) Keeping group members informed<br>48) Acting as a liaison	
**Evaluating change**	
Assessment<br>49) Performing/assisting with evaluation<br>50) Linking evidence implementation to patient outcomes<br>51) Acknowledging success, recognizing and celebrating achievements	

Source: Dogherty EJ, Harrison MB, Baker C, Graham ID: **Following a natural experiment of guideline adaptation and early implementation: a mixed-methods study of facilitation.** *Implementation Science* 2012
