# Supplementary material for: External facilitators and interprofessional facilitation teams: a qualitative study of their roles in supporting practice change
Source: Implement Sci. 2016 Jul 16;11:97. doi: 10.1186/s13012-016-0458-7 (PMC4947272; doi:10.1186/s13012-016-0458-7)
Supplement: Supplementary file 2 — Facilitation role categories and roles, by fields of expertise. PDF file displaying all 72 facilitation roles identified in this study, by category and theme, and how they relate to four fields of expertise (change management, project management, meeting management, and group/interpersonal dynamics). (PDF 361 kb) [file 13012_2016_458_MOESM2_ESM.pdf]

Additional file 2: Facilitation role categories and roles, by fields of expertise

Lessard S, Bareil C, Lalonde L, Duhamel F, Hudon E, Goudreau J, Lévesque L: **External facilitators and interprofessional facilitation teams: a qualitative study of their roles in supporting practice change**

A) Implementation-oriented facilitation roles			
	Fields of expertise		
Roles Themes	*Change management (CM)*	*Combination of CM+PM*	*Project management (PM)*
**A1. Legitimation of change/project**	a) Highlighting a need for change [1]<br>b) Displaying and generating enthusiasm about the project [1]<br>c) Emphasizing the benefits of change for people involved as opposed to poor practice as reason for change [1]	d) Assisting with/performing a formal/informal audit to gather baseline data [1]<br>e) Interpreting baseline data and providing feedback/insight into performance gaps [1]<br>f) Selecting area(s) for change relevant to staff/recognized as a priority [1]	
**A2. Formation of a powerful guiding coalition** [4]	a) Presenting change team members to the organization	b) Identifying a leader [1]<br>c) Advocating for resources and change [1]	
**A3. Communication of vision of change** [4] **and project guidelines**	a) Mentoring and role-modelling project-related practices [1]<br>b) Using storytelling	c) Knowledge translation/dissemination (assisting with conducting literature searches, obtaining articles, appraising and summarizing the evidence) [1]<br>d) Assembling/distributing information about vision and strategies<br>e) Helping to interpret the project [1]	
**A4. Empowerment of others to act towards change** [1, 4]	a) Helping to build in the structures/processes to support staff and help them overcome obstacles [1]<br>b) Creating local ownership of change [1]<br>c) Networking [1]<br>d) Assisting with adapting project requirements to the local context [1]<br>e) Increasing awareness of and helping overcome resistance to change [1]<br>f) Boundary-spanning (addressing organizational systems/culture), managing the different requirements of each discipline/role [1]	g) Establishing and allocating roles/delegating responsibilities [1]<br>h) Providing resources/tools for change [1]<br>i) Sharing ideas across project sites<br>j) Providing advice/guidance/assistance [1]<br>k) Problem-solving and addressing specific issues [1]<br>l) Bringing problems reported off-meeting back to the group<br>m) Acting as a liaison [1]<br>n) Providing skills training [1]<br>o) Being available as needed [1]<br>p) Meeting actors of change outside regular meetings<br>q) Establishing political links with / influencing stakeholders	
**A5. Application of PDSA cycle (plan-do-study-act)** [5, 6]		a) Stimulating critical inquiry and assisting group to develop/refine specific project-related questions [1]<br>b) Goal-setting and assisting with development of an action plan [1]<br>c) Helping identify and determine solutions to address potential barriers to project/change implementation [1]<br>d) Considering the bigger picture (impact on other unit sites, legal aspects, etc.)<br>e) Thinking ahead in the process [1]	m) Leading the project<br>n) Ensuring group remains on task and things are not missed [1]<br>o) Discussing specific cases/experiences within the scope of the project<br>p) Linking implementation actions to outcomes [1]<br>q) Keeping group members informed [1]

Legend: Inspired/adapted from [1] Dogherty *et al*. (2012), [2] Heron (1999), [3] Kolb *et al*. (2008), [4] Kotter (2007), [5] Nagykaldi *et al*. (2005), [6] Young (2009)

A) Implementation-oriented facilitation roles			
		f) Gathering information and assembling/distributing reports and materials [1]<br>g) Providing regular communication (emails, phone calls) [1]<br>h) Performing/assisting with evaluation about implementation aspects [1]<br>i) Making changes to the developed plan as necessary [1]<br>j) Taking on specific tasks [1]<br>k) Asking for/acknowledging feedback about implementation<br>l) Providing feedback about implementation	
**A6. Consolidation and continuity of improvements** [4]		a) Reflecting/planning on the "after-project" to consolidate improvements and institutionalize changes [4]	

B) Support-oriented facilitation roles			
**Roles Themes**	***Fields of expertise***		
	Meeting management (MM)	***Combination of MM+GID***	***Group and interpersonal dynamics (GID)***
**B1. Management of effective meetings**	a) Organizing/scheduling meetings [1]<br>b) Preparing meeting agenda and minutes<br>c) General administrative planning [1]<br>d) Leading/participating in meetings [1]<br>e) Performing meeting evaluations	f) Tailoring/adapting facilitation services to the local setting [1]<br>g) Encouraging/ensuring adequate participation during meetings [1]<br>h) Establishing ground rules [3]<br>i) Assigning meeting maintenance roles<br>j) Promoting consensus-building (shared decision-making) [1]	k) Listening actively, clarifying and summarizing the information
**B2. Enablement of relationship building** [1]			a) Welcoming/engaging new members in the team<br>b) Building relationships [1]
**B3. Handling of individual/group emotional life** [2]		a) Reporting/Managing conflict	b) Creating an open, supportive, and trusting environment conducive to change [1]<br>c) Observing group members' behaviors<br>d) Providing ongoing support/reassurance and constructive feedback [1]
**B4. Encouragement**			a) Maintaining momentum and enthusiasm [1]<br>b) Acknowledging ideas and efforts [1]<br>c) Encouraging effective teamwork [1]<br>d) Sharing benchmarking results from multiple sites to encourage team<br>e) Acknowledging success, recognizing and celebrating achievements [1]
**B5. Enablement of individual/group development** [1]			a) Providing education, training, or coaching to develop individuals or group

Legend: Inspired/adapted from [1] Dogherty *et al*. (2012), [2] Heron (1999), [3] Kolb *et al*. (2008), [4] Kotter (2007), [5] Nagykaldi *et al*. (2005), [6] Young (2009)
