# Supplementary material for: External facilitators and interprofessional facilitation teams: a qualitative study of their roles in supporting practice change
Source: Implement Sci. 2016 Jul 16;11:97. doi: 10.1186/s13012-016-0458-7 (PMC4947272; doi:10.1186/s13012-016-0458-7)
Supplement: Supplementary file 3 — Definitions and evidence related to facilitation roles themes. PDF file displaying descriptions and definitions of facilitation themes, as well as illustrating excerpts from TRANSIT study. (PDF 249 kb) [file 13012_2016_458_MOESM3_ESM.pdf]

Additional file 3: Definitions and evidence related to facilitation roles themes

Lessard S, Bareil C, Lalonde L, Duhamel F, Hudon E, Goudreau J, Lévesque L: **External facilitators and interprofessional facilitation teams: a qualitative study of their roles in supporting practice change**

The following will define and illustrate how each theme of the facilitation roles was reflected in TRANSIT.

A) Themes of implementation-oriented facilitation roles

Legitimation of change/project (A1)

Legitimation has been associated with the successful implementation of changes/projects [1–3]. It is about how far participants or stakeholders perceive the change/project as being right, proper or justified [3, 4]. Facilitation roles linked to the legitimation of the change/project reflect the activities or actions put forward in order to achieve an understanding of the need for change among change actors and stakeholders.

Excerpts related to TRANSIT:

"*We held a meeting [because they were questioning their participation in the project] and I said, 'In that case, why are you still continuing with this project?' ... It was to make them see ... the main reason why they agreed to participate in this project.*" (Interview T3-EF2 on IFT2)

"*I know how hard Doctor L has worked on selling this project. ... we invested a lot of time in trying ... to bring the doctors to an understanding, but they were very afraid of getting involved.*" (Interview T12-IFT1)

Formation of a powerful guiding coalition (A2)

Kotter combines the notions of having the necessary power to make change happen, and encouraging the group to work together, into a single step that he calls "forming a powerful guiding coalition" [1:99]. Because we divided implementation- and support-oriented facilitation roles, this theme only covers roles linked with the power aspect. The formation of a powerful guiding coalition therefore includes roles aimed at recognizing the change agents as leaders of the change effort.

Excerpts related to TRANSIT:

"*Also, what helps in this team, is that the pharmacist is a very good leader. ... He is a leader, but others participate in leadership too.*" (Interview T3-EF2 on IFT3)

"*Doctor L was thrilled about the kinesiologist and the role she will be assuming with patients. He wants us to organize a meeting with the doctors from his clinic, so they will feel confident about referring patients to the kinesiologist.*" (EF field notes-T3-FMG1)

Communication of vision and project guidelines (A3)

Communicating the vision is recognized as a key factor for the success of change/project implementation [1, 5]. It involves relaying change-related information in every possible way, as well as showing by example [1]. Facilitation roles within this theme are aimed at communicating the vision or "direction in which an organization needs to move" [1:99], by using techniques such as mentoring and storytelling, or by providing an understanding of the project through information on research protocol, project organization, new tools, evidence used, etc.

Excerpts related to TRANSIT:

"*If we hadn't had a facilitator, I don't think we would have worked towards these objectives… We wouldn't have had any direction, in fact. So I think that her role was really to define what the research was about.*" (Interview T6-IFT4)

"*Regarding the community pharmacists that work outside [the clinic] … many of those that work for the same company as me have come to me… asking for details, but again, it's all about communication… We did the letter probably a little late, since many of them had already seen patients with health booklets before they received any explanation. I gave them a maximum of information, but it's not always easy… they ask many questions about the project, it requires having enough time to clearly explain to everyone.*" (Interview T6-IFT4)

Empowerment of others to act towards change (A4)

Although "empowering group members" was seen as a single facilitation activity by Dogherty *et al*. [6], we have found that this theme encompasses a large number of facilitation roles. Similarly to Kotter's description of empowering others to act [1], these facilitation roles are aimed at removing obstacles to change, changing structures and processes as needed, creating local ownership, and allowing change actors to move forward towards change/project implementation.

Excerpts related to TRANSIT:

"[discussing motivational interviewing, which is one of the practices promoted by TRANSIT] *[The nurse] gives me examples of what she does, and asks me if she is being too directive. I reassure her, give further explanations, reinforcement, advice, and realignment. She will try to work on her approach.*" (EF Field notes T3-FMG2)

"*His peers told him: 'I don't see … where the nurse leaves her notes regarding TRANSIT… We are not going to search the whole file to find her sheet.' … [So] during that second meeting, Doctor G brought up the question: 'How are we going to identify the tools and documents*

related to TRANSIT?' ... The pharmacist said: *'Color codes.'... The nurse* said: *'It's a good idea, we will use color codes.' And she chose a flashy pink."* (Interview-T3-EF on IFT3)

Application of the PDSA (plan-do-study-act) cycle (A5)

Although Young considered "planning" and "preparation" separately, he identified "do-check-act" as one of the main themes recurrent across his change literature review, describing it as keeping the transition aligned and steering towards what needs to be delivered [7]. Since we found that all four steps of Deming's well-known PDSA cycle were emerging and looping throughout TRANSIT's implementation process, we decided to keep them together under this theme. The loop concept is recognized in project management (re: planning loops [8]), as well as in change management, especially with regard to the learning organization approach (i.e. single-loop and second-loop learning) [7, 9]. Used by practice facilitators [10] and other change agents, the PDSA cycle is a method that helps to structure improvement projects [11]. Facilitation roles under this theme are aimed at setting up plans (e.g. project management plan, schedules, budget plans, communications), steering and managing implementation efforts, monitoring progress and verifying the success or failure of actions implemented, and making adjustments as needed.

Excerpts related to TRANSIT:

"*A table that showed monitoring indicators was presented to the team. [External facilitator 1] asked everyone to collaborate in order to collect data and complete the report. ... ACTION: [The administrative assistant] will complete the data from February 15 to August 31. After that, the various professionals will all do their own monthly compilation.*" (Minutes T12-FMG1)

"*Doctor C suggests that before the end of the meeting we make a list of actions and assign these actions to those concerned. A list of actions was sent later that day to all IFT members.*" (Minutes T6-FMG4)

Consolidation and continuity of improvements (A6)

One of the concerns raised by interprofessional facilitation teams was how they could keep the project going after external facilitators left, or ensure improvement results were maintained. Kotter highlights the importance of keeping the process alive, as well as ensuring leadership succession [1]. The facilitation role under this theme is aimed at making sure that the positive outcomes of the change/project are maintained even after the facilitation team has dissolved or the facilitator has left (meaning that he/she "passed the baton" [12]).

Excerpts related to TRANSIT:

"*[The nutritionist] suggests brainstorming about the next steps. ... Questions to address: Do we apply the new practices to new patients? How should patient selection be done… [should we] broaden the criteria—choose our own criteria?*" (Minutes T12-FMG3)

"*After that I introduced the work we have started in order to build a project sustainability plan.*" (EF field notes-T12-FMG4)

B) Themes of support-oriented facilitation roles

Management of effective meetings (B1)

This theme has to do with creating a context that allows dialogue [13]. It includes facilitation roles aimed at making meetings happen (e.g. reaching participants to schedule meetings, sorting out logistics), structuring the meetings, and guiding the group through the agenda in respect of all individuals.

Excerpts related to TRANSIT:

"*We asked [External facilitator 2] to include… in her agenda, … a schedule with times… and when we get sidetracked, the person who manages the time [timekeeper] calls us to order, reminding us when we only have five minutes left to discuss an item.*" (Interview T6-IFT2)

"*I appreciate what she [the external facilitator] sends by email… she talks about the agenda, invites us to add other agenda items … She also sends a reminder and confirms the meeting.*" (Interview T6-IFT4)

Relationship development (B2)

Relationship development is one of the aspects that groups must go through during their first step of development, often called the "forming" step [14]. Facilitation roles concerning relationship development are mostly aimed at building bridges between group members [15], but they can also occur with different actors involved with implementing the change/project. These roles are represented by activities such as each person around the table giving a presentation, preparing/sharing documents with members' contact details, running team-building activities, or meeting new members prior to their first meeting.

Excerpts related to TRANSIT:

"*Doctor D welcomes everyone and suggests that we go around the table so everyone can introduce themselves. What is mentioned: function, years within family medicine group, brief description of provided services (for some), exchanging business cards.*" (Minutes T6-FMG2)

"*For [their] first meeting, I let the discussions flow a bit more, so the people could get to know each other, talk about their concerns, their research-related experience, and learn about where they come from and what they did in the past.*" (EF field notes-T3-FMG4)

Handling of individual/group emotional life (B3)

"Emotion is to do with the fulfilment or frustration of our individual needs and interests in the forms of joy, surprise, anger, grief and so on" [16]. Facilitation roles related to this theme are aimed at creating and maintaining an open, supportive, and trusting environment; detecting the emotions expressed by change actors (verbally or non-verbally), listening to their concerns and reacting accordingly; as well as dealing with conflicts that may be roadblocks to change/project implementation (e.g. gathering opinions, verifying level of agreement, holding meetings with individuals and in groups to resolve the conflict, etc.).

Excerpts related to TRANSIT:

"*She was lacking a lot of self-confidence and self-esteem. Of course, she always called me if she had relevant questions/reflections about TRANSIT, but as a facilitator, I could not avoid these aspects, because one of my roles is to support team members, individually and as a group, […] without falling into a psychologist-patient relationship*." (EF Field notes T12-FMG2)

"*Because I had felt the discomfort before the start of the meeting–[the nutritionist] had told me rapidly, and there were discussions between group members… I could hear their different comments and they were not smiling much…– [so at the start of the meeting] I said 'I think you have certain comments about the new budget distribution?'*" (EF Field notes T9-FMG2)

Encouragement (B4)

Encouraging can take different forms, including praising, agreeing, valuing the contribution of others, demonstrating a warm and solidary attitude, showing understanding towards one's point of view, and accepting ideas or suggestions [17]. These facilitation roles are expected to provide the encouragement to work together and achieve goals, through positive reinforcement and appreciation.

Excerpts related to TRANSIT:

"*[The nurse] is a leader, she really helped us to hang in there. … I personally had given up and said, 'I can't do it anymore.' … 'Don't quit, N [admin], we'll get there.' That's [the nurse] who said that, and she was the one who was in over her head.*" (Interview T12-IFT2)

"*The whole team congratulates the nurses and support staff for their effectiveness, because the objective of 100 patients seems to have been reached, according the most recent information received by [the external facilitator]*." (Minutes T6-FMG2)

Enablement of individual/group development (B5)

Facilitation has been associated with training and group development ever since Lewin's T-groups started in the mid-1940s [18]. The facilitation role under this theme is aimed at educating, training, and coaching individuals and/or groups on: quality-improvement philosophy, methods,

and skills [19, 20]; values supported by the change/project; and group- or individual-development-related subjects, tools, and techniques (e.g. facilitation, leadership, teamwork, problem-solving).

Excerpt related to TRANSIT:

"*The PDSA cycle was briefly explained during the meeting. I presented the model to them, which will allow us to evaluate the processes and activities that we will integrate into their practices.*" (EF Field notes-T3-FMG4)

"[Regarding more advanced training on motivational interview] *[The nutritionist] gathered information on next training dates and cost.*" (Minutes T12-FMG2)

References in Additional file 3:

1. Kotter JP. Leading change. *Harvard Business Review* 2007, 85:96-103.
2. Pasmore WA. Tipping the balance: Overcoming persistent problems in organizational change. In *Research in Organizational Change and Development. Volume* 19: Emerald Group Publishing Limited; 2011: 259-292
3. Rondeau A. L'évolution de la pensée en gestion du changement : leçons pour la mise en œuvre de changements complexes. *Téléscope* 2008, 14:12.
4. Tyler TR. Psychological perspectives on legitimacy and legitimation. *Annual Review of Psychology* 2006, 57:375-400.
5. Andersen ES, Birchall D, Jessen SA, Money AH. Exploring project success. *Baltic Journal of Management* 2006, 1:127-147.
6. Dogherty EJ, Harrison MB, Baker C, Graham ID. Following a natural experiment of guideline adaptation and early implementation: a mixed-methods study of facilitation. *Implementation Science* 2012, 7:9.
7. Young M. A meta model of change. *Journal of Organizational Change Management* 2009, 22:524-548.
8. Stackpole Snyder C. A user's manual to the PMBOK guide [internet]. 5th ed. Hoboken (NJ): John Wiley & Sons; 2013 [cited 2014 Aug 27]. Available from Safari books online.
9. Argyris C. *On organizational learning.* 2nd ed. Malden, MA: Blackwell Publishers; 1999.
10. Nagykaldi Z, Mold JW, Aspy CB. Practice facilitators: A review of the literature. *Family Medicine* 2005, 37:581-588.
11. Van Assen M, Van Den Berg G, Pietersma P. *Key management models: The 60+ models every manager needs to know.* 2nd ed. Harlow, UK: Pearson Education; 2009.
12. Looney JA, Shaw EK, Crabtree BF. Passing the baton: Sustaining organizational change after the facilitator leaves. *Group Facilitation* 2011, 11:15-23.
13. Senge PM. *The fifth discipline: the art and practice of the learning organization.* United States of America: Currency Doubleday; 2006.
14. Smith G. Group development: A review of the literature and a commentary on future research directions. *Group Facilitation* 2001, 3:14-45.
15. Clawson VK, Bostrom RP, Anson R. The role of the facilitator in computer-supported meetings. *Small Group Research* 1993, 24:547-565.
16. Heron J. The complete facilitator's handbook [internet]. London: Kogan Page; 1999 [cited 2013 April 16]. Available from Books24x7.
17. Benne KD, Sheats P. Functional Roles of Group Members. *Group Facilitation* 2007, 8:30-35.
18. Keltner JS. Facilitation catalyst for group problem solving. *Management Communication Quarterly* 1989, 3:8-32.
19. Cook E, Dale BG. Organizing for continuous improvement: An examination. *The TQM Magazine* 1995, 7:7-13.
20. Knox L, Taylor EF, Geonnotti K, Machta R, Kim J, Nysenbaum J, Parchman M. Developing and running a primary care practice facilitation program: A how-to guide [internet]. Rockville (MD): Agency for Healthcare Research and Quality; 2011 [cited 2013 April 3] Report No.: 12-0011. Available from: http://pcmh.ahrq.gov/sites/default/files/attachments/Developing_and_Running_a_Primary_Care_Practice_Facilitation_Program.pdf
