# Supplementary material for: External facilitators and interprofessional facilitation teams: a qualitative study of their roles in supporting practice change
Source: Implement Sci. 2016 Jul 16;11:97. doi: 10.1186/s13012-016-0458-7 (PMC4947272; doi:10.1186/s13012-016-0458-7)
Supplement: Supplementary file 4 — Facilitation roles by change agent and relationships involved in facilitation. PDF file displaying facilitation roles undertaken by EFs and by IFTs, as well as change actors involved for each facilitation role. (PDF 181 kb) [file 13012_2016_458_MOESM4_ESM.pdf]

Additional file 4:

Facilitation roles by change agent and relationships involved in facilitation

Lessard S, Bareil C, Lalonde L, Duhamel F, Hudon E, Goudreau J, Lévesque L: **External facilitators and interprofessional facilitation teams: a qualitative study of their roles in supporting practice change**

Table legend: Ref. Axx : Implementation-oriented facilitation roles

Ref. Bxx : Support-oriented facilitation roles

Italics: Emerging role

- ■ Role directed towards the IFT
- ◆ Role directed towards the Family Medicine Group
- ❖ Role directed towards other external change agents (e.g. clinicians, health agencies, support groups)
- ● Role directed towards one or the other EF
- 🗎 Role directed towards the Research Group

		CHANGE AGENT UNDERTAKING FACILITATION ROLE	
**Ref. in add. file 2**	**FACILITATION ROLES**	**EF**	**IFT**
A1a	Highlighting a need for change	■◆❖	◆❖
A1b	Displaying and generating enthusiasm about the project	■	■
A1c	Emphasizing the benefits of change for people involved as opposed to poor practice as reason for change	■	■◆
A1d	Assisting with/performing a formal/informal practice audit to gather baseline data	🗎	■
A1e	Interpreting baseline data and providing feedback/insight into performance gaps	■	◆
A1f	Selecting an area for change relevant to staff/recognized as a priority	■	■
A2a	*Presenting change team members to the organization*	◆	◆
A2b	Identifying a leader	■	■
A2c	Advocating for resources and change	■ 🗎	■ ◆
A3a	Mentoring and role-modelling project-related practices	■ ◆	◆ ❖
A3b	*Using storytelling*		◆
A3c	Knowledge translation/dissemination (assisting with conducting literature searches, obtaining articles, appraising and summarizing the evidence)	■	■ ◆
A3d	*Assembling/distributing information about vision and strategies*	■ 🗎 ❖	◆❖
A3e	Helping to interpret the project	■ ◆ ❖	■ ◆ ❖
A4a	Helping to build in the structures/processes to support staff and help them overcome obstacles	■ ❖	■ ◆ ❖
A4b	Creating local ownership of change	■	◆
A4c	Networking	■ ❖	■ ❖
A4d	Assisting with adapting project requirements to the local context	■	● ■ ◆
A4e	Increasing awareness of and helping overcome resistance to change	■ ◆ ❖	● ◆ ❖
A4f	Boundary-spanning (addressing organizational systems/culture), managing the different requirements of each discipline/role	■	◆
A4g	Establishing and allocating roles/delegating responsibilities	■	■ ◆
A4h	Providing resources/tools for change	■	● ■ ◆
A4i	*Sharing ideas across project sites*	■	■
A4j	Providing advice/guidance/assistance	● ■ ◆❖	● ■

Table legend: Ref. Axx : Implementation-oriented facilitation roles

Ref. Bxx : Support-oriented facilitation roles

Italics: Emerging role

■ Role directed towards the IFT
◆ Role directed towards the Family Medicine Group
❖ Role directed towards other external change agents (e.g. clinicians, health agencies, support groups)
● Role directed towards one or the other EF
🗎 Role directed towards the Research Group

CHANGE AGENT UNDERTAKING FACILITATION ROLE

**Ref. in add. file 2**	**FACILITATION ROLES**	**EF**	**IFT**
A4k	Problem-solving and addressing specific issues	■ ◆ 🗎	● ■
A4l	*Bringing problems reported off-meeting back to the group*	■	◆
A4m	Acting as a liaison	■ ◆ 🗎 ❖	● ■ ◆❖
A4n	Providing skills training	■	
A4o	Being available as needed	■ ◆	■
A4p	*Meeting actors of change outside regular meetings*	■ ◆❖	■ ◆
A4q	*Establishing political links with/influencing stakeholders*	❖	❖
A5a	Stimulating critical inquiry and assisting groups to develop/refine specific project-related questions	■	
A5b	Goal-setting and assisting with development of an action plan	■	■ ◆
A5c	Helping identify and determine solutions to address potential barriers to project/change implementation	● ■ 🗎	■ ◆
A5d	*Considering the bigger picture (impact on other unit sites, legal aspects, etc.)*	● ■	❖
A5e	Thinking ahead in the process	■	
A5f	Gathering information and assembling/distributing reports and materials	■ 🗎 ❖	● ■ ◆❖
A5g	Providing regular communication (emails, phone calls)	● ■ ◆ 🗎 ❖	■
A5h	*Performing/assisting with evaluation about implementation aspects*	■	■ ◆
A5i	Making changes to the developed plan as necessary	■	■
A5j	Taking on specific tasks	■ ❖	■
A5k	*Asking for/acknowledging feedback about implementation*	■	■ ◆
A5l	*Providing feedback about implementation*	■ 🗎	● ■ ◆
A5m	*Leading the project*	■	■ ◆
A5n	Ensuring group remains on task and things are not missed	■	● ◆
A5o	*Discussing specific cases/experiences within the scope of the project*		■ ◆
A5p	Linking implementation actions to outcomes		■ ◆
A5q	Keeping group members informed	■	■ ◆❖
A6a	*Reflecting/planning on the "after-project" to consolidate improvements and institutionalize changes*	■ ◆	■ ◆
B1a	Organizing/scheduling meetings	● ■ ◆ 🗎	● ■ 🗎
B1b	Preparing meeting agenda and minutes	■	●
B1c	General administrative planning	■	
B1d	Leading/participating in meetings	■ ◆ 🗎	● ■ ◆
B1e	*Performing meeting evaluations*	■	
B1f	Tailoring/adapting facilitation services to the local setting	■	

Table legend: Ref. Axx : Implementation-oriented facilitation roles

Ref. Bxx : Support-oriented facilitation roles

Italics: Emerging role

- ■ Role directed towards the IFT
- ◆ Role directed towards the Family Medicine Group
- ❖ Role directed towards other external change agents (e.g. clinicians, health agencies, support groups)
- ● Role directed towards one or the other EF
- 🗎 Role directed towards the Research Group

		**CHANGE AGENT UNDERTAKING FACILITATION ROLE**	
**Ref. in add. file 2**	**FACILITATION ROLES**	**EF**	**IFT**
B1g	Encouraging/ensuring adequate participation during meetings	■	■
B1h	*Establishing ground rules*	■	■
B1i	*Assigning meeting maintenance roles*	■	■
B1j	Promoting consensus-building (shared decision-making)	● ■	■
B1k	*Listening actively, clarifying and summarizing the information*	■	
B2a	*Welcoming/engaging new members in the team*	■	■
B2b	Building relationships	■ ◆	■ ◆
B3a	*Reporting/Managing conflict*	■ ◆ 🗎	●
B3b	Creating an open, supportive, and trusting environment conducive to change	■	● ■ ◆
B3c	*Observing group members' behaviors*	■	
B3d	Providing ongoing support/reassurance and constructive feedback	● ■ ◆	● ■
B4a	Maintaining momentum and enthusiasm	■	■
B4b	Acknowledging ideas and efforts	■	● ■
B4c	Encouraging effective teamwork	■	■
B4d	*Sharing benchmarking results from multiple sites to encourage team*	■	
B4e	Acknowledging success, recognizing and celebrating achievements	■	■
B5a	Providing education, training, or coaching to develop individuals or group	■	
